# Where Is the Action in Perception? An Exploratory Study With a Haptic Sensory Substitution Device

**DOI:** 10.3389/fpsyg.2020.00809

**Published:** 2020-04-28

**Authors:** Tom Froese, Guillermo U. Ortiz-Garin

**Affiliations:** ^1^Embodied Cognitive Science Unit, Okinawa Institute of Science and Technology Graduate University, Okinawa, Japan; ^2^Laboratory 25, Department of Experimental Psychology, Faculty of Psychology, National Autonomous University of Mexico, Mexico City, Mexico

**Keywords:** active perception, embodied cognition, agency, perceptual discrimination, enactive perception, Enactive Torch, volition, active touch

## Abstract

Enactive cognitive science (ECS) and ecological psychology (EP) agree that active movement is important for perception, but they remain ambiguous regarding the precise role of agency. EP has focused on the notion of sensorimotor invariants, according to which bodily movements play an instrumental role in perception. ECS has focused on the notion of sensorimotor contingencies, which goes beyond an instrumental role because skillfully regulated movements are claimed to play a constitutive role. We refer to these two hypotheses as *instrumental agency* and *constitutive agency*, respectively. Evidence comes from a variety of fields, including neural, behavioral, and phenomenological research, but so far with confounds that prevent an experimental distinction between these hypotheses. Here we advance the debate by proposing a novel double-participant setup that aims to isolate agency as the key variable that distinguishes bodily movement in active and passive conditions of perception. We pilot this setup with a psychological study of width discrimination using the Enactive Torch, a haptic sensory substitution device. There was no evidence favoring the stronger hypothesis of constitutive agency over instrumental agency. However, we caution that during debriefing several participants reported using cognitive strategies that did not rely on spatial perception. We conclude that this approach is a viable direction for future research, but that greater care is required to establish and confirm the desired modality of first-person experience.

## Introduction

The fields of enactive cognitive science (ECS) and ecological psychology (EP) are two prominent alternatives to orthodox cognitive science, and which are in agreement about the need for a relational account of mind situated at the personal level (Chemero, 2009). They also share a commitment to the claim that perception is a dynamic process, and hence that movement is essential for perception, yet they also disagree on a number of points regarding the nature of perception (Varela et al., 2017; Heras-Escribano, 2019). It is still unclear whether these disagreements are signs of deeper conceptual differences, or are merely differences in emphasis, which highlights the need of establishing a closer dialog (Lobo, 2019). One major point of contention is the precise role of agency in the perceptual process. More specifically, it is still an open debate to what extent action makes a difference to perception and perceptual learning, i.e., whether it matters if bodily movements are self-initiated, actively regulated, and/or intentionally guided, or merely accidentally caused by the agent’s body, or even completely environmentally driven.

Ecological psychology started as a non-representational account of perception (Gibson, 1979), but has since developed into a more comprehensive non-representational psychology. As such, it also has a strong interest in agency and active exploration (Heras-Escribano, 2019). Yet, arguably, it has most famously focused on the experimental study of *perceptual invariants* (Mossio and Taraborelli, 2008), which are arguably independent of the source of perceptual change. In fact, some do not require any bodily movement at all. For example, when EP uses optic flow to derive time-to-contact it does not matter whether perceptual flow is brought about by bodily movements actively performed by the perceiver, or if flow is just passively undergone due to changes in the perceiver’s environment (i.e., produced “by object *R* as it moves toward the eye,” Chemero, 2009, p. 124). More generally, EP does not distinguish between: (1) optical changes due to intentional self-movement, e.g., human locomotion, (2) optical changes due to accidental self-movement, e.g., being hurled towards a collision, and (3) optical changes due to environmental movement, e.g., an approaching ball to be intercepted; all of these changes can be captured by the same invariant of optic flow because it is mathematically defined independently of agency, namely as the rate of acceleration of optical expansion (Lobo et al., 2018). Research into active, dynamic or effortful touch may seem to be provide a counterexample, but even here a key hypothesis is that the perceptual capabilities are defined in terms of detection of invariance in the patterns of tissue deformation (Carello and Turvey, 2015); the source of the deformation is irrelevant for the shape of the patterns. We will consider active touch in more detail below.

To be fair, following Gibson, most classical, and contemporary research in EP strongly emphasizes the importance of action and agency for perception and human experience (e.g., Gibson, 1979; Reed, 1982; Käufer and Chemero, 2015). Nevertheless, it is also fair to say that the focus of interest has been on the other direction of influence, namely on the claim that actions can be controlled by perception of affordances, like catching an approaching baseball. It is sufficient for our argument that both kinds of claims tend to be compatible with an instrumental interpretation of the role of active movement in perception. Thus, bodily movement is an important, but not exclusive, manner of generating optic flow and detecting time to contact. The upshot of this instrumental role, whereby e.g., the explanatory weight is placed directly on the rate of optical expansion, is that EP – its many claims to the contrary notwithstanding – is still partially aligned with the orthodox “input-output picture” (Hurley, 1998). At this stage, it remains unclear how perception would differ when its invariants are instantiated for reasons other than self-movement. We refer to this compatibility with an instrumental role of self-movement as the hypothesis of *instrumental agency*. This leads to the experimental prediction that perception should be unaffected by whether the perceiver is actively exploring an object or undergoing the same changes passively.

Enactive cognitive science, on the other hand, has famously focused on the role of action in perception (Noë, 2004; O’Regan et al., 2005; Myin, 2016; Di Paolo et al., 2017; Froese and González-Grandón, 2019), which foregrounds the role of the perceiver’s skillful capacity for regulating movement in the constitution of perceptual experience. One key concept here is that meaningful perception depends on the perceiver’s exercise of their mastery of *sensorimotor contingencies* (O’Regan and Noë, 2001), i.e., of the regular ways in which sensations would change as a consequence of bodily movements. The major approaches to ECS differ in the details of how this dependence on the exercise of mastery should be conceived (Bishop and Martin, 2014), e.g., in terms of metacognition, intentional directedness, or adaptive regulation, but they share a common hypothesis of *constitutive agency*. Although it is not exactly clear how perception during active vs. passive movement conditions would differ, the prediction is that the perceptual experience will be affected in some way. For example, we might expect there to be a difference in the qualitative feel of the experience (O’Regan, 2011), there might be an attenuation in its felt significance (Di Paolo et al., 2017), or an impaired sense of object presence (Noë, 2012). As such, ECS goes beyond just EP’s instrumental role of bodily movement and forms an important part of the broader class of *action-based* theories of perception (Briscoe and Grush, 2017).

Proponents of EP often make claims that also favor the stronger hypothesis of constitutive agency, and it would be interesting if EP developed those intuitions in a more explicit manner. We hope that the kind of psychological study we will propose can facilitate this process.

## Previous Work

Experimental evidence often cited by EP and ECS in support of the importance of self-movement typically comes from two major classic sources on perceptual learning and more recent versions:

(1) the “kitten carousel” studies initiated by Held and Hein (1963), which concluded that passive exposure to optic flow is not sufficient for the ontogenetic development of normal visual perception, and
(2) the “sensory substitution” studies initiated by Bach-y-Rita et al. (1969), which concluded that exposure to prerecorded time series of sensory stimuli is not sufficient for the lifetime learning of normal visual perception.

A key issue with source (1) is that it is problematic to derive strong claims about the quality of perceptual experience based on an animal behavioral result. According to Prinz (2006), it is equally conceivable that the kittens from active and passive conditions had exactly the same visual experiences, but that the kittens from the passive condition had not yet had the opportunity to acquire an adequate mapping of that visual experience to motor commands. In other words, it is still possible to formulate an interpretation of the results that is consistent with the orthodox input-out picture.

Held and Hein’s study was replicated and extended by Walk et al. (1988). They added two new passive conditions: one in which the kittens’ attention to visual stimuli was enhanced by being able to control the automated movement of their own cart, and another in which the kitten’s cart remained immobile but was placed in front of a more dynamic environmental spectacle involving moving toy cars. Even though these kittens were unable to use their legs to self-locomote, their legs responded appropriately to the visual cliff test. The authors explained these results in terms of EP and argued that what is important is attention to perceptual variation, but not whether locomotion is self-initiated. Nevertheless, kittens in all conditions were still capable of self-initiating movements of their heads and eyes, and hence they could in fact actively explore sensorimotor contingencies in this restricted visuomotor domain. In other words, it is equally conceivable that the kittens were sufficiently motivated to acquire mastery of these available visuomotor contingencies.

Advances in technology have permitted much more sophisticated versions of this paradigm. For example, a recent study placed pairs of mice in a virtual reality setup akin to the kitten carousel (Attinger et al., 2017). Each mouse was placed on a large trackball in front of a screen with the head fixed in position. Whenever the active mouse walked its display would change accordingly, while the other mouse’s trackball and display would change identically, forcing it to undergo a similar visuomotor loop but without being able to actively influence the visual stimulation. The authors analyzed recordings of neural activity from primary visual cortex (V1) and found that coupling between motor output and visual feedback is necessary for the functional development of visual processing. This result seems to favor constitutive agency. However, even though the trackballs rotated identically, mice in the uncoupled condition were able to move differently, and hence were exposed to highly irregular sensorimotor invariances and sensorimotor contingencies. It is therefore not surprising that their perceptual skills developed poorly. Finally, although differences in development of neural activity in V1 are suggestive, it is not clear in general how such neural differences are related to visual experience (Hurley and Noë, 2003).

Two common problems with these animal studies are that it is difficult to isolate agency, and also to derive claims about perceptual experience from behavioral and/or neural data.^1^ A more promising approach for the scientific study of the role of active movement in perceptual experience are psychological studies involving participants that can give reports about how changes in conditions affect their first-person experience (Froese et al., 2012b). This brings us to second classic source.

In particular, the use of sensory substitution interfaces provides a useful experimental technique for simplifying and controlling human sensorimotor loops (Bach-y-Rita and Kercel, 2003; Lenay et al., 2003; Froese et al., 2012a). Such studies consistently find that performance on perceptual tasks is improved when the changes in participant’s sensations are contingent on their own movements (e.g., Bach-y-Rita, 2002; Auvray et al., 2005; Díaz et al., 2012). However, none of these sensory substitution studies has been able to address the confound that was also an issue for the mouse virtual reality study by Attinger et al. (2017): uncoupling sensory stimulation from passive participants’ movements makes it impossible for self-initiated movement to influence sensation, but at the same time it scrambles the regularities inherent in sensorimotor invariances and sensorimotor contingencies more generally. It is therefore unclear whether it is the lack of sensorimotor regularity or the lack of agency which causes the impaired performance.

In summary, so far these lines of research have been unable to arbitrate between the two hypotheses with respect to the role of agency. What is needed is an experimental approach that ensures that both active and passive participants undergo identical sensorimotor loops, involving the same sensations and movements, but in such a way that only the active participant can freely regulate the movements. This is necessary so that any difference in performance can then be attributed to the difference in participant’s active involvement in movement (Richardson et al., 2000). It also remains to be seen if any differences seen during perceptual learning would also still apply to mature perception.

A fitting starting point is touch because it is one of the most active modalities, although experimental results are not always consistent with this impression (Symmons et al., 2004). This ambiguity is likely related to the fact that control conditions are often not strict enough. For instance, a study of discrimination of arm movement distances found that active movement is associated with greater precision (van Beek et al., 2014). However, the passive condition induced constant arm movement rather than replicating actual movement patterns, and hence the authors’ conclusion that taking away agency from tool operators would deteriorate precision is not necessarily warranted.

Another study removed this confound by replicating patterns of sensorimotor flow: an active participant manipulated a haptic stylus (a Phantom device) to categorize one of four different kinds of 3D geometric shapes, while at the same time a passive participant held onto another Phantom device that underwent the same movements and generated the same feedback (Symmons et al., 2005). In this way, even the perception of the location and movement of body parts, known as proprioception or kinesthesia, is also largely kept the same across participants. It was found that active participants tended to be more accurate, but there was no statistically significant difference. This result could be related to the fact that passive participants were still relatively active: although they did not control the direction of movement, they still had to actively grasp the stylus and follow its trajectory in a compliant manner. To increase passivity, it would be better if the passive condition involved no effort of movement at all.

In this brief research report, we describe a novel version of this kind of double-participant setup that satisfies this stricter control condition of passivity for the first time. We also present the results of an exploratory study of width discrimination using this setup.

## Materials and Methods

**FIGURE 1:**
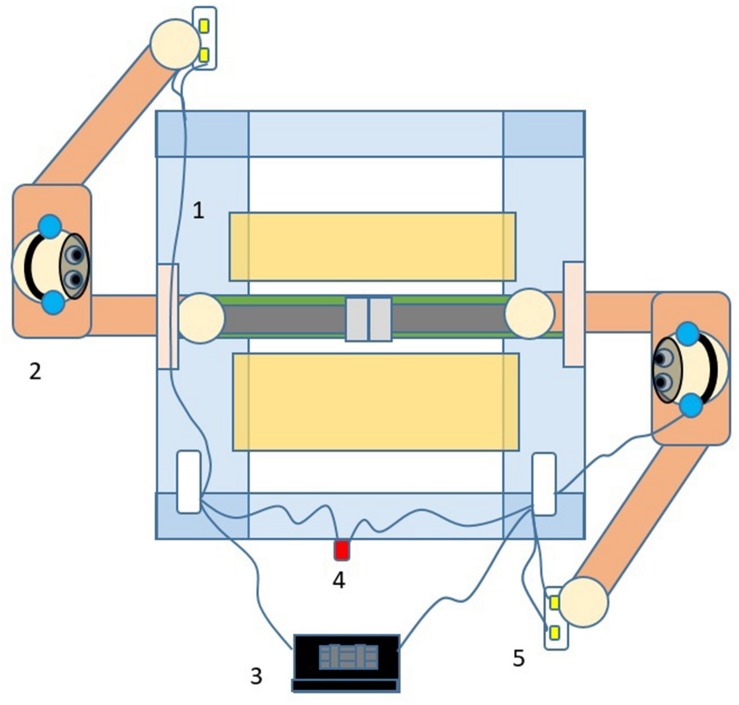
Illustration of the double-participant experimental setup. Several elements of the setup are highlighted in the illustration. *1:* The experimental box, which is illustrated in more detail in Figure 2 below. *2:* One of the participants with opaque goggles and noise-canceling headphones to restrict perception to the tactile modality. The dominant hand grasps an Enactive Torch inside the box. *3:* Laptop computer for data recording. *4:* Button for experimenter to start the next trial. *5:* Circuit box to capture participant responses.

**FIGURE 2:**
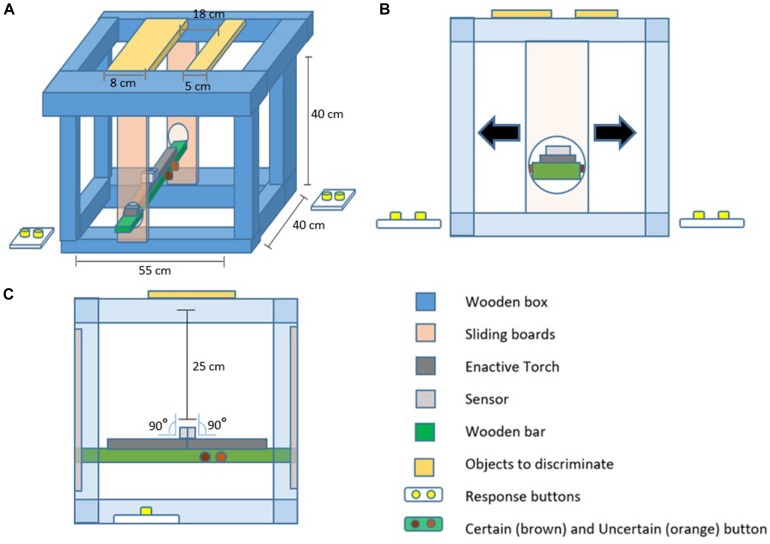
Schematics of the experimental box. Several details are highlighted in color and described in the figure’s legend. Panels show different perspectives on the box. **(A)** Illustration of the dimensions of the box. Note that both Enactive Torch sensory substitution devices are attached on top of the same wooden bar that connects the two vertical sliding boards, thereby ensuring that both participants undergo the same horizontal displacement and receive the same vibrotactile feedback. Each participant’s end of the wooden bar also featured a set of two buttons on the side; the button closer to the sensor head indicated confidence in the discrimination response provided with the button box held in the left hand. **(B)** Close-up of a participant’s side of the box. Circular cavities were made to the sliding boards so that participants could reach in to grasp their Enactive Torch. Arrows indicate possibility of horizontal displacement. **(C)** Close-up of side of the box showing that the sensor heads of the Enactive Torch was oriented upwards at 90°. This prevented interference between the two devices and enabled detection of target objects placed on top of the box.

**FIGURE 3:**
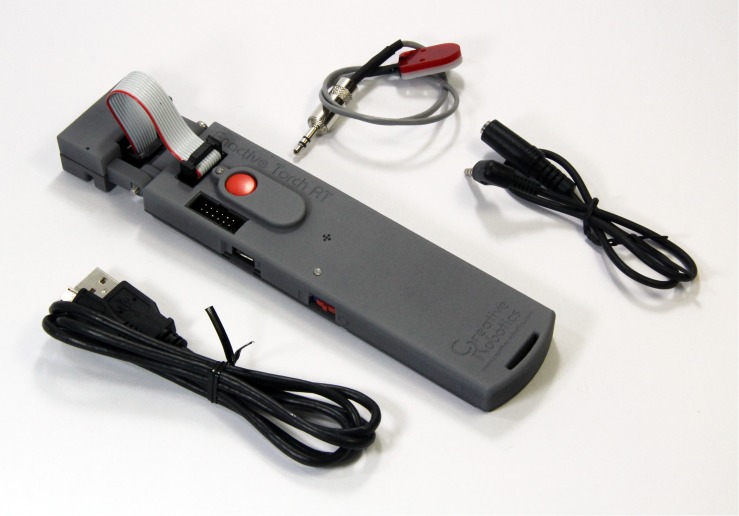
Photo of the Enactive Torch Research Tool (ETRT). We made use of ETRT v1.0. Note that for this study we turned the sensor head upwards at a 90°C angle so that subjects were given vibrotactile sensations corresponding to the objects placed on top of the experimental box, as illustrated in Figures 2, 3. The photo also shows the data cable and a cable with a small actuator and its extension cable for external vibrotactile output. For this study we transferred data to the laptop computer via Bluetooth and employed the vibrating actuator built into the ETRT itself.

The double-participant setup was implemented with a custom-made experimental box consisting of mechanical and electronic components (see Figures 1, 2 for details). Pairs of participants were seated at opposite sides and could undergo the same movements (albeit in a mirrored direction) and changes in vibrotactile sensations at the same time. The sensations were mediated by a hand-held sensory substitution device called the *Enactive Torch* (Figure 3; Froese et al., 2012a), which translates infrared-based measures of distance to nearby objects into intensity of vibrotactile feedback in the user’s hand. Like a cane for blind people, this device permits people to learn to perceive passages through objects in space (Favela et al., 2018), and user’s walking trajectories coincide with those of visually-guided locomotion (Lobo et al., 2019).

The crucial methodological advantage of working with the Enactive Torch is that, by mediating object perception through a fixed sensory substitution device, we could more easily ensure that both participants underwent exactly the same sensorimotor loop. Minor differences in proprioception cannot be ruled out because it encompasses a complex of sensations that includes muscle force and effort (Taylor, 2009), which will necessarily differ across active and passive participants.

The task was inspired by a recent study in EP on width discrimination that involved the Enactive Torch (Favela et al., 2018). In our study, participants had to discriminate between the widths of two objects, and then to indicate which one was the wider one and to indicate if this was a confident discrimination. The two objects were 5 cm and 8 cm wide, which resulted in roughly 70% correct responses after data normalization. This level of discrimination difficulty was chosen as a value between chance level (50%) and potential ceiling effects (100%).

### Participants

In total, we tested 70 participants (32 men and 38 women; mean age = 21.48, SD = 2.59) combined into 35 pairs. All participants were recruited from our research group and students’ networks of contacts at the National Autonomous University of Mexico (UNAM). They volunteered to participate without financial reward and signed informed consent forms. All participants were right-handed, and all reported no psychiatric or motor disorders.

### Procedure

Each participant of a test pair was randomly assigned to one of two groups: passive or active. They were unaware of this assignment, and while they could guess that another person was being tested in the same room, they remained unaware of the fact that they were connected in a pair. Participants were blindfolded and then one-by-one guided into the experimental room, where they were seated on a chair, on one side of the wooden box. The side was randomly assigned. They wore headphones that played noise in the background to avoid distraction of sounds; we chose brown over white noise because participants prefer lower frequencies of sound masking (Hongisto et al., 2015). Then the participants underwent a brief training procedure, which consisted in the following steps:

1. One participant removed their headphones.
2. They were then guided to grasp their respective Enactive Torch. They were also familiarized with the two sets of buttons to indicate the wider object and their level of confidence.
3. All the instructions were given. A similar but subtly different instruction was given depending on the group, as explained below.
4. Two test trials were run, involving two objects with 3 cm difference in width.
5. The participant was given an opportunity to ask questions.
6. The participant puts on their headphones again.

Then the other participant underwent the same training steps. Regarding the specific instructions, for the active group, the instructions were to grasp the Enactive Torch with their dominant hand and to move it horizontally (right to left or vice-versa) at a constant speed for a particular period to sense the width of the two objects. The duration of each trial was 5 s. Participants were to start moving when they heard a beep, and when they heard the beep again, they were to stop and click to indicate which of the two objects was the wider one. They also clicked on one of the other two buttons next to the Enactive Torch to indicate their level of confidence. They then returned the Enactive Torch to the starting point before the next trial could begin.

For the passive group, the instructions were to rest their dominant hand on the Enactive Torch, and to avoid any resistance to the movements that were going to be produced after the beep. They were also told that when the same tone rang again, the device was going to come to rest, and they should perform the required button clicks. Then the bar would return to its starting point.

No feedback was provided during the experiment. In total, 120 trials were run per pair of participants. However, for the current research question, only the first 60 trials were analyzed given that the second set of 60 trials involved a different condition. At the end of every experiment, we individually asked the participants about the strategies they used to solve the task.

### Data Analysis

Statistical analysis of task performance was carried out using the software *R* released by the R Core Team (2019). We first obtained the proportion of correct answers for every participant, and then obtained the descriptive statistics of the proportions for each group. We ran a two-tailed paired *t*-test analysis to compare the proportions of correct answers for both groups. The null hypothesis was that there are no differences between the average percentages of correct responses between groups.

## Results

The responses recorded for all trials can be found in Supplementary Datasheet S1. The descriptive statistics of the proportion of correct answers is summarized in Table 1.

**TABLE 1:** Summary of descriptive statistics.

	**Group**	**Mean**	**Median**	**Std. dev.**	**Min.**	**Max.**
Correct responses (correct vs. incorrect)	Active	0.708	0.717	0.102	0.500	0.917
	Passive	0.721	0.721	0.081	0.517	0.867
Confident responses (certain vs. uncertain)	Active	0.680	0.7	0.126	0.433	1
	Passive	0.731	0.733	0.149	0.383	0.983

*The active and passive groups were compared in terms of the proportion of correct answers to the width discrimination task.*

No statistically significant differences were found among the active and passive groups in terms of the proportions of correct responses [*t*(34) = -0.74734, *p* = 0.46], nor of confident responses [*t*(34) = -1.4639, *p* = 0.1524]. Therefore, actively initiated and regulated sensorimotor loops and passively undergone sensorimotor loops led to the same proportion of correct answers.

Informal debriefing interviews after each experiment revealed that some participants had used a counting strategy to solve the width discrimination task. For example, several reported that they would start counting at the start of the first phase of vibrotactile feedback until the end of that phase, do the same for the second phase of feedback, and then compared the counts to determine which object took longer to be traversed. This strategy was aided by the fact that many active participants chose to move slowly within the 5 s limit of a trial. In other words, for these participants width discrimination performance was not based on tactile space perception.

## Discussion

The null result is more in line with the more conservative hypothesis of instrumental agency, rather than with the stronger hypothesis of constitutive agency. However, in hindsight the experimental setup still needs to be improved in several respects.

• *Attentional load*. Poorer performance during the active condition could have resulted from interfering effects of increased attentional and cognitive load, which have been attributed to decisions about how to move (Richardson et al., 1981). Conversely, reduction of cognitive load in active conditions has been associated with a relative increase in haptic discrimination performance (Richardson et al., 2006).
• *Cognitive strategies*. Several participants reported a cognitive (counting and comparing) strategy, which implies that they did not actually perceive width. If so, then the passive group was not necessarily disadvantaged; to the contrary, being moved enabled them to focus their attention on the cognitive strategy. It is possible that the training was not sufficient for perceptual learning, thereby forcing participants to rely on a cognitive strategy.
• *Potential movement*. The ECS theory of sensorimotor contingencies only requires *overt* movement for the learning or acquisition of mastery, but not for the subsequent exercise of that mastery, which also works with *potential* movement (Myin, 2016). Future work should record muscle and/or neural activity in order to try to detect the implicit exercise of mastery of sensorimotor contingencies (Froese and González-Grandón, 2019). Alternatively, the passive condition could involve a mechanical device that fixes the participant’s arm and forces it to reproduce exactly the same movement pattern as the active condition, but this is more difficult to implement than the double-participant setup.
• *Degrees of freedom.* The active group might have been overly constrained, which leveled the playing field with the passive condition. This was done to ensure that all trials were comparable across participants. Future work in this direction will have to learn to embrace the possibilities of open-ended exploration and the individual variability that this will generate. In particular, it may be necessary to consider tasks that permit the spontaneous transition between several possible stable patterns of behavior (Dotov et al., 2019).
• *From ends to means*. Normally perception is a means to an end, but in our task perception was the end itself. Again, this may have invited more cognitive strategies. It would therefore be preferable to turn perceptual discrimination into a function at the service of a higher-level action goal (Favela et al., 2018).

More generally, future work in this direction needs to pay greater attention to whether participants are learning to solve the task by incorporating the sensorimotor mediation afforded by the sensory substitution device into a genuinely perceptual experience (Schumann and O’Regan, 2017). This points to a crucial methodological problem: how to better assess participants’ experience of using a sensory substitution interface (Kałwak et al., 2018). As revealed in this study, good performance on a perceptual task is not sufficient to discriminate between perceptual and cognitive strategies. And while simple subjective reports can aid in making coarse-grained categorizations, it remains to be seen how we can obtain more fine-grained distinctions. For instance, it is conceivable that ECS and EP will come to agree that, after learning, sensorimotor invariants are sufficient for objectively discriminating *what* is perceived – e.g., time-to-contact (instrumental agency), and that active self- movement then only makes a difference for the subjective experience of *how* it is perceived – e.g., the conscious feel of vision (constitutive agency), for example with respect to the richness of its presence. However, the details still need to be worked out and operationalized. Moreover, tracking differences in “what it is like” is precisely the problem of consciousness and calls for specialized first-person methods (Petitmengin et al., 2019). Accordingly, despite repeated claims that active movement is essential, there is still more theoretical and experimental work to be done to determine precisely where is the action in perception.

## Data Availability Statement

The complete datasets for this study are available in the Supplementary Material.

## Ethics Statement

All participants read and signed a consent form. The experimental protocol was approved by the Department of Experimental Psychology of the Faculty of Psychology at the National Autonomous University of Mexico – UNAM.

## Author Contributions

TF conceived of the general idea to test the role of action in perception with a double-participant setup using the Enactive Torch sensory substitution interface, and he wrote this manuscript. GO-G conceived of and piloted the experimental protocol, designed and built the experimental box, recruited the participants, conducted the experiment, and analyzed the results. TF and GO-G finalized the manuscript together.

## Conflict of Interest

The authors declare that the research was conducted in the absence of any commercial or financial relationships that could be construed as a potential conflict of interest. The reviewer DD declared a past co-authorship with one of the authors, TF, to the handling editor.

## Footnotes

1 These problems are also shared by agent-based simulation models that are inspired by the hypothesis of constitutive agency (e.g., Suzuki et al., 2005; Buhrmann et al., 2013). It is unclear whether synthetic approaches can capture the relevant aspects of agency and experience (Froese and Taguchi, 2019).

## Supplementary Material

DATA SHEET S1 Task performance – participants’ responses for all trials analyzed in this article.
